# Bioactive Compounds as Modulators of N-Formyl Peptide Signaling in Chronic Diseases

**DOI:** 10.3390/molecules30142981

**Published:** 2025-07-16

**Authors:** Livia Alvarenga, Ludmila F. M. F. Cardozo, Márcia Ribeiro, Fernanda Kussi, Marta Esgalhado, Denise Mafra

**Affiliations:** 1 Department of Cardiopneumology, Faculty of Medicine, University of São Paulo (FMUSP), São Paulo 01246-903, SP, Brazil; 2 Graduate Program in Nutrition Sciences, Fluminense Federal University (UFF), Niterói 24020-140, RJ, Brazil; ludmilacardozo@id.uff.br; 3 Graduate Program in Cardiovascular Sciences, Fluminense Federal University (UFF), Niterói 24070-090, RJ, Brazil; 4 Graduate Program in Biological Sciences—Physiology, Federal University of Rio de Janeiro (UFRJ), Rio de Janeiro 21941-902, RJ, Brazil; marciaribeiro@biof.ufrj.br; 5 Department of Basic Pathology, Federal University of Paraná, Curitiba 81531-980, PR, Brazil; fernandakussi@ufpr.br; 6 CBIOS—Universidade Lusófona’s Research Center for Biosciences and Health Technologies, 1749-024 Lisbon, Portugal; marta.esgalhado@ulusofona.pt

**Keywords:** N-formyl peptides, formyl peptide receptors, bioactive compounds, diet, non-communicable disease

## Abstract

In physiological situations involving cell damage, molecules derived from mitochondria or bacteria are produced. These molecules are known as N-formyl peptides and are detected by formyl peptide receptors (FPRs), which stimulate immune cells to migrate to the specific site of injury or infection. Despite their initially beneficial effects on health, N-formyl peptides also contribute to the development or exacerbation of chronic non-communicable diseases. Therefore, understanding the metabolic pathways related to the involvement of N-formyl peptides and FPRs may increase our ability to regulate immune responses and precisely target FPRs with personalized strategies, offering a promising approach for the treatment of specific diseases. In this way, bioactive compounds in food may influence N-formyl peptides, interacting with the receptors either competitively or by inhibiting them, which affects the inflammatory response and oxidative reactions of cells. This review examines the pathways associated with forming N-formyl peptides, the activation of FPRs, and the roles of bioactive compounds in regulating N-formyl peptides.

## 1. Introduction

N-formyl peptides constitute a distinct group of molecules characterized by the addition of a formyl group to the amino group of the N-terminal methionine residue. Examples include the N-formyl-methionyl-leucyl-phenylalanine (fMLP) and phenol-soluble modulins (PSMs) produced by *Staphylococcus aureus* [1,2]. Additionally, mitochondria can also produce formylated peptides, such as the N-termini of NADPH dehydrogenase subunits 4 and 6, which are released during cellular damage [1].

N-formyl peptides are potent agonists of formyl peptide receptors (FPRs), a subfamily of G protein-coupled receptors (GPCRs) primarily located on immune cells, including monocytes and neutrophils, which mediate cellular chemotaxis [3]. Upon activation, FPRs initiate a series of intracellular signals that induce morphological changes in immune cells and promote the release of either pro- or anti-inflammatory cytokines, highlighting the dual roles of these receptors in immune modulation [1].

Deregulated responses of FPRs can lead to several diseases, including inflammatory process disorders [4]. Targeting FPRs is emerging as a promising strategy to modulate immune responses, with the development of agonists or antagonists that hold potential for conditions where the precise control of immune activation is crucial. Furthermore, this proposal highlights that bioactive compounds in food possess essential antioxidant and anti-inflammatory properties [5,6]. Therefore, it is plausible that dietary interventions could be welcomed as adjuvant nutritional strategies in treating diseases, with a focus on regulating N-formyl peptides [7].

This narrative review of N-formylated peptides examines the pathways involved in their synthesis, the activation of their receptors, and their roles in the gut microbiota, inflammation, and oxidative stress. It also presents an overview of their involvement in chronic diseases. Additionally, this literature review aims to clarify how bioactive compounds regulate N-formyl peptides and FPRs.

## 2. N-Formyl Peptides and Their Receptors

**Figure 1:**
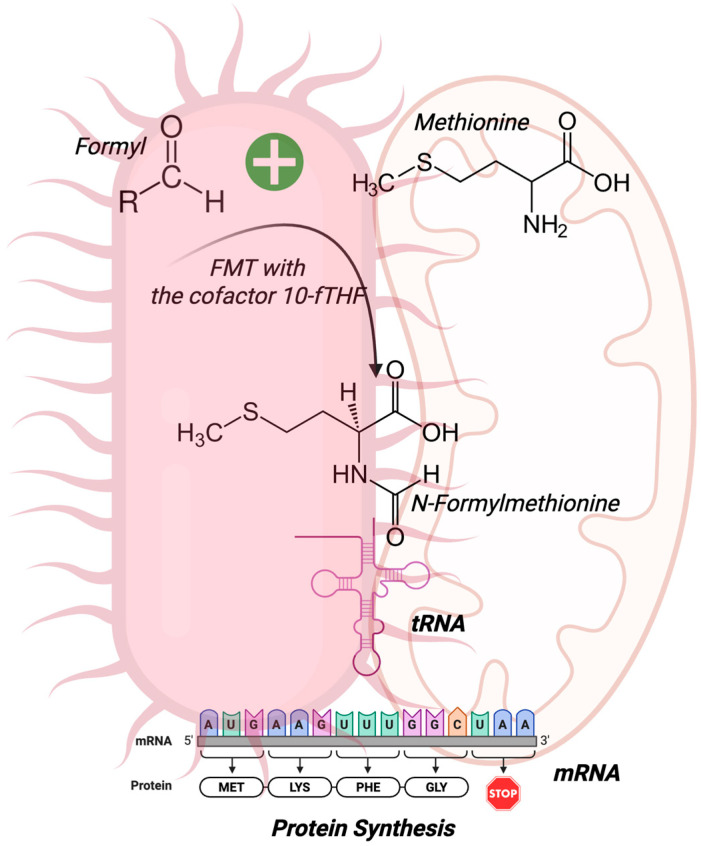
N-Formylmethionine (fMet) synthesis. fMet is the first amino acid in the synthesis of all bacterial proteins. The enzyme formyltransferase (FMT) and its cofactor 10-formyltetrahydrofolate (10-fTHF) originate from fMet, and the complex mRNA and fMet-tRNA initiate protein synthesis. Due to their bacterial ancestry, mitochondria are also a source of N-formyl peptides. Created by BioRender.com.

In 1964, while studying the translation process in bacteria, Marcker and Sanger discovered that the N-terminal portion of proteins can be formylated, resulting in the compound N-formylmethionine (fMet), which the authors hypothesized to be an essential initiator of protein synthesis [8]. Two years later, Adams and Capecchi confirmed this, verifying that fMet initiates the synthesis of bacterial proteins [9] and is encoded by the AUG initiation codon [10]. The enzyme formyltransferase (FMT) carries out the addition of the formyl group to the α-amino of methionine using 10-formyltetrahydrofolate (10-fTHF) as a cofactor [11] (Figure 1).

Most of the methionines in the amino-terminal portion are removed by methionine aminopeptidase (MetAP), as most bacterial proteins have alanine, serine, or threonine as their initial amino acids, rather than methionine [12]. This cleavage typically occurs when the second amino acid in the sequence has a small side chain, specifically, one with a gyration radius of 1.29 Å or less, which allows proper access and activity of the MetAP enzyme [13].

Excision of the N-terminal methionine occurs before protein folding, resulting in a diversity of amino acids in this position [14]. According to Sandikci et al. [14], electrostatic interactions associate peptide deformylase (PDF) and MetAP with the ribosomal surface [14]. PDF is the first to bind through binding sites at the exit of the ribosome tunnel as polypeptides emerge by performing deformylation (removal of the N-terminal formyl group) [15]. Finally, the MetAP enzyme interacts with the ribosome at a location that overlaps the PDF binding position, thereby removing methionine from the nascent protein chains [14].

It has been believed that fMet initiates polypeptide synthesis in all bacteria. According to Mader et al., protein formylation is essential for bacterial metabolic activities such as the anaerobic degradation of arginine [16,17]. Indeed, the inactivation of the FMT gene in *Staphylococcus aureus* impaired folic acid-dependent metabolic pathways. However, it is not yet known what cellular implications are caused by the absence of methionine formylation in the initial peptide portion found in some bacteria [17].

It is crucial to note that fMet is not exclusive to bacteria. The initiator of protein synthesis has also been found in eukaryotic organelles descended from bacteria, such as mitochondria [18] and chloroplasts [19]. Mitochondrial N-formyl peptides are produced during the translation of mitochondria-encoded proteins, starting with an N-formylmethionine, a process similar to that in bacterial protein synthesis [20].

Mitochondria are crucial for various metabolic processes in the human body, including ATP production, regulation of cell death, oxidative signaling, the innate immune response, calcium balance, fatty acid metabolism, and autophagy [21]. Mitochondrial damage-associated molecular patterns (DAMPs) express molecular signatures related to mitochondrial N-formyl peptides (FMIT) that act on FPRs [16]. According to the literature, mitochondrial DAMPs activate neutrophils via FPR and p44/42 mitogen-activated protein kinase (MAPK) [16].

During cellular stress or mitochondrial damage provoked by trauma, inflammation, and infections, N-formyl peptides are released, and they have been implicated in the induction of inflammatory processes through the activation of neutrophils via the FPR1 and FPR2 receptors. This activation precedes the rapid infiltration of neutrophils into injured tissues, followed by the production of chemokines by the injured tissues [4]. Furthermore, FMIT has been implicated in sepsis-like syndrome and cardiovascular collapse. The activation of FPRs by host-derived or formylated bacterial peptides is crucial for the local control of infections and wound healing [22]. However, releasing formylated peptides into the bloodstream, which is evident in conditions such as sepsis or severe trauma, can lead to several harmful effects [16].

FPRs are chemoattractant receptors in the GPCR family. They are integral to the immune system’s defense mechanisms and inflammatory responses. Structurally, FPRs consist of polypeptide chains with several distinct regions: an extracellular N-terminal domain, multiple intracellular and extracellular loops, and seven transmembrane helices that connect to an intracellular C-terminal domain. These structural components enable FPRs to interact with various ligands and transduce signals crucial for the migration and function of immune cells [3].

Three genes code for the receptors, including FPR1 and FPR2, which are expressed in leukocytes, monocytes, macrophages, and natural killer cells. In addition, they can also be found in non-immune cells, including smooth muscle cells, lens epithelial cells, fibroblasts, astrocytes, hepatocytes, microvascular endothelial cells, and neuroblastoma cells [2,23]. FPR3 is found in monocyte-derived macrophages, mature dendritic cells, and tissue-specific macrophages [1]. It is essential to highlight that FPR1, FPR2, and FPR3 are receptors expressed in humans, whereas mice have distinct isoforms with different responses to ligands such as fMLF. Therefore, caution is necessary when extrapolating the results from rodent models to human physiology, especially regarding the effects of FPR-targeting agonists and antagonists [5].

The activation of FPRs originates from detecting and binding to pathogen-associated molecular patterns (PAMPs) and DAMPs such as fMLP and other N-formylated peptides [1]. Although fMLP is a classical agonist of both FPR1 and FPR2, its activity is most potent at FPR1. fMLP has over a thousand-fold larger binding to FPR1 compared to FPR2, due to differences in the electrostatic charges of the binding site and the ability to accommodate short peptides. This functional selectivity implies that fMLP-mediated inflammatory effects occur predominantly through FPR1, supporting its role as a key mediator in rapid inflammatory responses [24].

After binding to FPR, the dissociation of G-protein α (Gα) from the G-protein βγ (Gβγ) subunit occurs. The latter activates phospholipase Cβ (PLCβ), which releases calcium from intracellular stores and activates protein kinase C (PKC). Another pathway triggered by the βγ subunit is that phosphoinositide-3-kinase gamma (PI3Kγ) activates PKCs and protein kinase B (Akt). Furthermore, the α subunit activates Ras superfamily GTPases, which in turn contribute to the activation of the MAPK, p38, and extracellular signal-regulated kinase (ERK) pathways [3].

**Figure 2:**
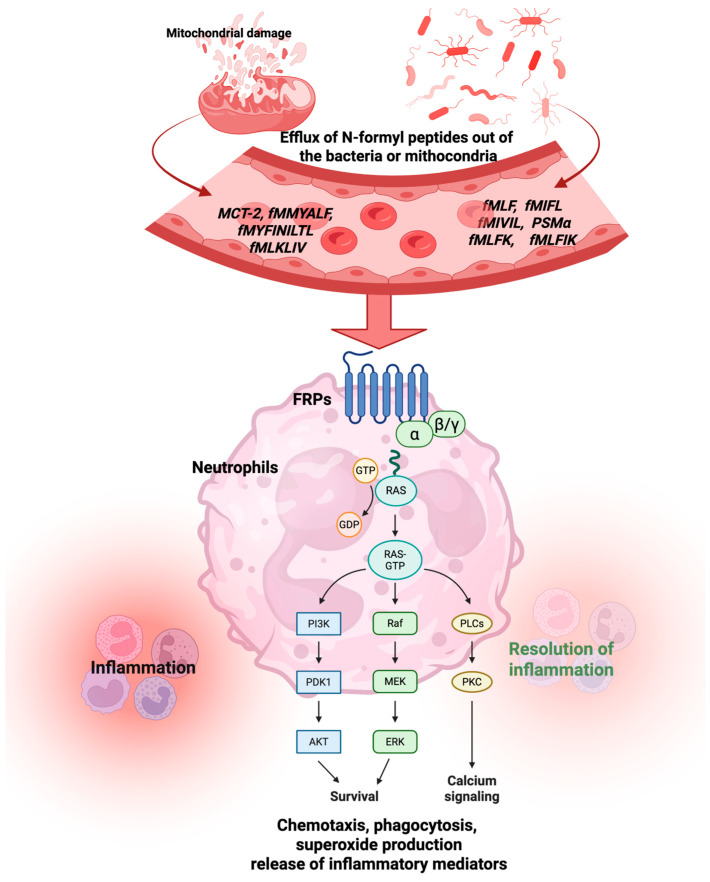
Activation of the FPR by formyl peptides from mitochondria or bacteria triggers both physiological and pathological processes. Abbreviations: fMLP, N-formyl-methionyl-leucyl-phenylalanine; fMIFL, N-formyl-methionine-isoleucine-phenylalanine-leucine; fMIVIL, N-formyl-methionine-isoleucine-valine-isoleucine-leucine; PSMα, modulins of the alpha type; fMLPK, N-formyl-Met-Leu-Phe-Lys; fMLPIK, N-formyl-Met-Leu-Phe-Ile-Lys; MCT-2, mitocryptide-2; fMMYALF, N-formyl-methionine-methionine-tyrosine-alanine-leucine-phenylalanine; fMYFINILTL, N-formyl-methionine-tyrosine-phenylalanine-isoleucine-asparagine-isoleucine-leucine-threonine-leucine; fMLKLIV, N-formyl-methionine-leucine-lysine-leucine-isoleucine-valine. Created by BioRender.com.

The activation of FPRs is associated with several physiological and pathological processes, including the activation of complements (C5a and C3a) and chemokines, which recruit and guide leukocytes to the site of bacterial infection and damaged tissues [25]. Lower doses of fMLP (5–6 nM) increase the expression of CD11b, CD62L, and CD66b in PMNs, whereas higher doses (19 and 50 nM of fMLP) boost the production of reactive oxygen species (ROS) [26]. Liu et al. discovered that the knockdown of FPR2 triggered an inflammatory response induced by lipopolysaccharide (LPS) in macrophages. They also demonstrated that LPS activated the nuclear factor kappa B (NF-κB) pathway in the lungs of mice and siCon RAW264.7 cells, resulting in increased expression of p-IKKα, p-I-κBα, and p-NF-κB (p65). The knockdown of FPR2 significantly reduced the phosphorylation of IKKα, I-κBα, and NF-κB, as well as the translocation of NF-κB to the nucleus. Moreover, in the LPS-induced model, the levels of the HO1, NQO1, and Nrf2 proteins were decreased, whereas in the Fpr2 knockout model, they returned to normal levels [27]. Human pulmonary microvascular endothelial cells (HPMVECs) treated with fMLP showed increased levels of tumor necrosis factor alpha (TNF-α), interleukin (IL)-6, IL-8, ROS, and malondialdehyde (MDA), along with decreased levels of SOD and reduced expression of Nrf2 and HO-1 [28]. Figure 2 shows the chemotaxis, phagocytosis, superoxide production, and release of inflammatory mediators provoked by FPR activation. Short N-formyl peptides, such as fMLP, are primarily generated through the proteolytic degradation of bacterial or mitochondrial proteins during infection or cellular damage (e.g., by neutrophil proteases) [1]. While theoretically any N-formylated peptide fragment could be produced, only a limited subset of sequences has been shown to act as potent chemotactic agents. Among them, fMLP is considered the prototypical and most potent agonist of FPR1, and has been extensively used as a reference ligand in experimental models [1].

In addition to N-formyl peptides, FPRs may also bind to other ligands, such as HIV envelope protein peptides, which utilize FPR2 as a co-receptor for viral entry. Furthermore, serum amyloid A (SAA) is a well-explored ligand for FPR2, particularly in cases of pneumonia. It exacerbates infection and acute lung inflammation. Serum amyloid A stimulates the production of metalloproteinase-1 and -3 and increases the activation of NF-κB and AP1 [4].

Conversely, Lipoxin A4 (LXA4, a lipid mediator derived from arachidonic acid) promotes the increase in the level of tissue inhibitor of metalloproteinase-2, which leads to the inhibition of NF-κB and AP-1 activation [29]. Similarly, Resolvin D1 (RvD1), a docosahexaenoic acid (DHA) polyhydroxy metabolite, binds to FPR2, helping to stop the migration of inflammatory cells and promote the resolution phase of inflammation, which aids in tissue repair and recovery. Through their interactions with FPR2, both LXA4 and RvD1 play pivotal roles in resolving inflammation, reducing tissue damage, promoting healing, and limiting leukocyte recruitment to the site of infection [30].

Additionally, Annexin A1 (ANXA1) plays a crucial role in cellular processes, including membrane repair. When ANXA1 binds to FPR2, it triggers a cascade of intracellular signaling events that inhibit neutrophil migration to inflammatory sites, thereby reducing the overall inflammatory response [31,32].

Furthermore, it is worth noting that FPRs can act as trans-activators of receptor tyrosine kinases (RTKs), thereby promoting the activation of common signaling pathways between these two systems through cross-signaling mechanisms [33]. Activation of GPCRs, such as FPRs, leads to the activation of effector proteins capable of inducing the phosphorylation of tyrosine residues in the cytoplasmic domain of RTKs. Secondary messengers produced in this process, such as ROS, can also directly activate these receptor tyrosine kinases [33].

Therefore, the significance of studying N-formyl peptides and FPRs, as well as their biological effects on the body, is emphasized [34]. Because they can interact with a wide range of pro- and anti-inflammatory ligands, they regulate diseases such as systemic sclerosis, amyloidosis, Alzheimer’s disease (AD), acquired immunodeficiency syndrome (AIDS), obesity, diabetes, and cancer [4,35,36].

## 3. N-Formyl Peptides in Pathological Conditions

**Figure 3:**
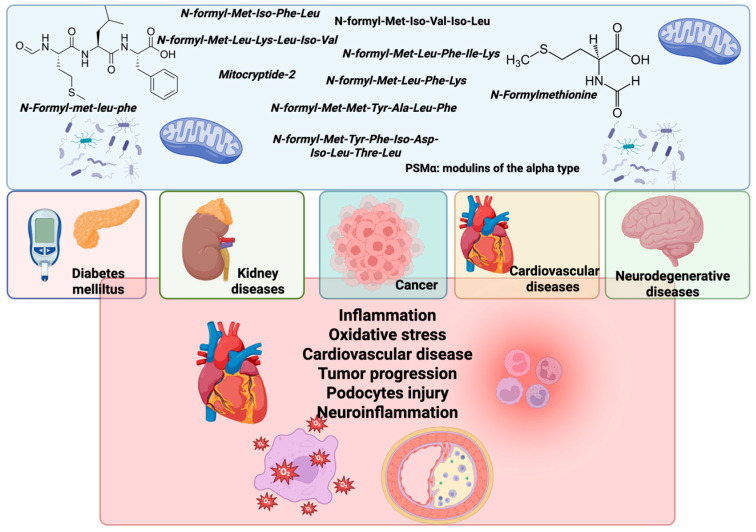
Impacts of mitochondria and bacteria N-formyl peptides on the progression of various pathological conditions. N-formyl peptides such as fMLP, as well as those derived from mitochondrial proteins, bacterial sources, and PSMα peptides, function as immunomodulatory agents. These peptides are involved in the pathogenesis of multiple diseases, including diabetes mellitus, kidney disorders, cancer, cardiovascular diseases (CVDs), and neurodegenerative diseases. The illustration highlights key pathological mechanisms associated with N-formyl peptide activity, including inflammation, oxidative stress, the progression of cardiovascular disease (CVD), tumor development, podocyte injury, and neuroinflammation. Created by BioRender.com.

Formyl peptides contribute to the progression of diabetes through insulin resistance, chronic inflammation, and beta-cell dysfunction [37]. They can also lead to glomerular damage, tubulointerstitial fibrosis, and proteinuria, thereby exacerbating chronic kidney disease [38]. These peptides contribute to neuroinflammation and neuronal injury, worsening neurodegenerative diseases and neurological harm [39]. N-formyl peptides promote inflammation and vascular dysfunction, contributing to the development of atherosclerosis, myocardial infarction, and hypertension. Formyl peptides can also enhance cancer progression [23,40] (Figure 3).

Additionally, several lines of evidence suggest that the activation of FPRs profoundly regulates cellular metabolism. For example, in lung carcinoma cells, signaling via FPR2 redirects glucose and glutamine uptake to anabolic pathways, stimulating the pentose phosphate pathway, pyrimidine nucleotide synthesis, and glutamine transport via ASCT2 [41,42]. In dendritic cells, the absence of FPR2 results in a reduced mitochondrial respiratory metabolic rate and decreased expression of genes in the Krebs cycle and respiratory chain. In neutrophils, activation by fMLP increases glutamine utilization, thereby promoting the generation of NADPH, which is essential for ROS production during the respiratory burst [43]. Furthermore, the activation of FPR2 stimulates the arachidonic acid pathway via phospholipase A2 and 5-lipoxygenase, increasing the release of pro-inflammatory eicosanoids [43,44]. Together, FPRs not only mediate chemotactic responses but also reprogram macronutrient and nucleotide metabolism to support immune and inflammatory responses [42].

### 3.1. Gastrointestinal Disorders

FPRs in non-phagocytic cell types, particularly intestinal epithelial cells (IECs), have garnered significant attention because bacterial formyl peptides have a profound influence on the immune response and gastrointestinal homeostasis [45]. The activation of FPRs by N-formyl peptides triggers immune responses within the gut, leading to the targeted migration of immune cells, such as neutrophils and macrophages, to sites of infection or inflammation [45].

In 1999, Gao et al. demonstrated the essential role of FPR1 in host defense mechanisms by revealing impaired neutrophil chemotaxis toward fMLP in Fpr1-null mice, which led to increased susceptibility to infection with monocytogenes and elevated mortality rates [46]. In *T. gondii*-infected Fpr1-null mice, the significance of neutrophils in preventing luminal cast formation and systemic bacterial translocation was evident [47]. In a colitis-induced model, fpr1-null mice exhibited decreased leukocyte migration into the inflamed gut [48]. Evidence suggests that fMLP stimulates FPR1 in the intestinal lumen [49].

In addition to their potent chemotactic properties, N-formyl peptides have been implicated in directly facilitating the elimination of pathobionts within the intestinal lumen by inducing the production of superoxide in neutrophils, triggering ERK phosphorylation, and enhancing cell proliferation and migration to promote wound closure and maintain intestinal homeostasis. Thus, formyl peptides can be considered potent antimicrobial agents, particularly at high concentrations, shielding against invasion by pathogens [50]. Additionally, the stimulation of FPR1 in IECs triggers a cytoprotective response characterized by Hsp27 expression and activation of MAPK pathways, including p38 and ERK1/2, which contribute to cellular protection, inflammatory modulation, the maintenance of epithelial integrity, and cell proliferation—all crucial for mucosal repair and the maintenance of colonic barrier integrity [30,51].

The depletion of FPR2 in a colitis-induced model resulted in substantial damage to the colonic epithelium, increased leukocyte infiltration, a reduced colonic crypt size, decreased mucin (MUC)2 production, and heightened bacterial invasion, particularly by *E. coli* [52]. Furthermore, Fpr2/3-null mice showed reduced monocyte recruitment to injury sites in the same colitis model, emphasizing their role in colonic mucosal wound repair [53].

The immune response orchestrated by N-formyl peptides not only influences microbiota dynamics but also significantly affects the growth and viability of various bacterial species, thereby shaping the overall microbial composition and function [30]. Neutrophil activation through FPR1 leads to the rapid depletion of microenvironmental oxygen via NOX2-mediated ROS generation while simultaneously increasing the expression of MUC3 in IECs. This promotes the enrichment of anaerobic bacterial consortia within damaged mucosal areas. Notably, *Akkermansia muciniphila*, a major member of this consortium, enhances mucosal wound repair through FPR1 activation and the induction of IEC-specific NOX1-dependent redox signaling, facilitating epithelial cell proliferation and migration—essential processes in mucosal healing. Furthermore, an analysis of the wound-associated microbiota reveals temporal shifts toward anaerobic taxa, which thrive in the oxygen-depleted environment and gradually diminish as the wound heals [54].

Recently, studies have shown that Fpr2-null mice have an altered gut microbial composition, characterized by decreased levels of Deferribacteres and Firmicutes, as well as increased levels of Proteobacteria, compared to their wild-type (WT) counterparts. The authors also revealed that in a colitis-induced model, Fpr2-null mice displayed a higher presence of *E. coli* in the colon, indicating that commensal *E. coli* may exhibit pathogenic traits, potentially as a compensatory response by the host [52].

**Figure 4:**
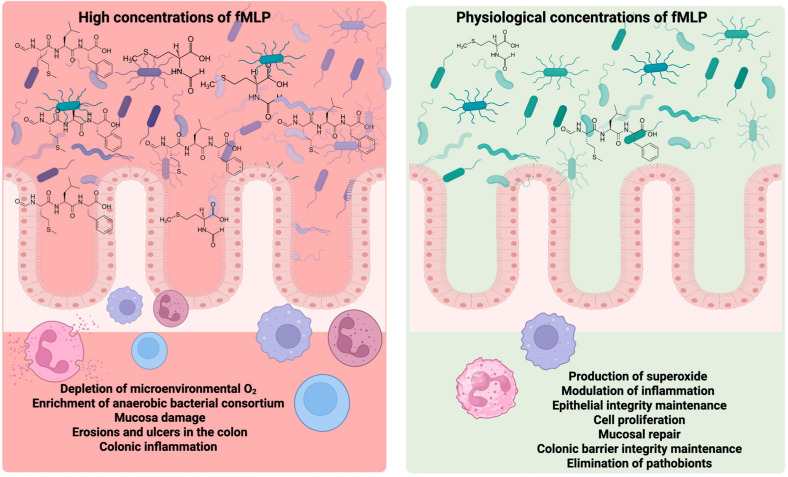
Dose-dependent effects of N-formyl-methionyl-leucyl-phenylalanine (fMLP) on intestinal homeostasis. At physiological concentrations, fMLP promotes superoxide production, modulates inflammation, maintains epithelial integrity, stimulates cell proliferation, supports mucosal repair, reinforces colonic barrier function, and facilitates the elimination of pathobionts—processes that collectively contribute to intestinal health. In contrast, elevated concentrations of fMLP are associated with adverse outcomes, including the depletion of microenvironmental oxygen, overgrowth of anaerobic bacterial consortia, mucosal damage, colonic erosion, ulcer formation, and inflammation. Created by BioRender.com.

Physiological concentrations of fMLP protect the intestinal epithelial barrier; however, high doses may lead to gut dysbiosis, characterized by changes in microbial diversity, composition, and function, which can result in various gastrointestinal disorders [47] (Figure 4).

### 3.2. Diabetes

Previous studies have shown that activation of the FPR1 receptor by N-formyl peptides, such as fMLP, can directly induce the translocation of glucose transporters (GLUTs). In Chinese hamster ovary cells transfected with FPR1, stimulation with fMLP promoted the mobilization of GLUT1 to the plasma membrane, resulting in a consequent increase in glucose uptake, suggesting a direct role of the GPCR in this process [55]. Similarly, it has been demonstrated that the stimulation of FPR1 is also capable of triggering GLUT4 translocation in an Akt-dependent manner, although independent of classical phosphatidylinositol 3-kinase isoforms [56].

In addition, Wang et al. (2025) highlight the dual nature of FPR2, which can mediate both pro- and anti-inflammatory responses, depending on the ligand and the cellular microenvironment [57]. This characteristic is especially relevant in diseases such as type 2 diabetes (T2D), marked by both chronic inflammation and failures in inflammatory resolution mechanisms. In models of diabetic retinopathy, for example, FPR2 activation has been linked to neuroglial dysfunction and pathological angiogenesis, suggesting its active involvement in the microvascular complications of the disease [37,57].

FPR2 activation has been linked to diabetic nephropathy due to its activation by the agonist human cathelicidin antimicrobial peptide (LL37), the induction of mitochondrial permeability transition pore (mPTP) opening, and increased mitochondrial ROS levels. These factors lead to the elevated production of neutrophil extracellular traps [58]. Inhibition of the LL37/FPR2/mPTP axis mitigates ROS production and cellular damage, making it a potential therapeutic target [58].

On the other hand, ANXA1 is a well-known endogenous anti-inflammatory ligand for FPR2 [59]. The ANXA1-FPR2 pathway plays a protective role in insulin resistance by inhibiting the phosphorylation of protein kinase C (PKC), thereby reducing the expression of inflammatory cytokines, such as IL-6 [60]. Hyperglycemia can decrease the levels of the pro-resolving lipid mediator RvD1 and downregulate RvD1 receptors, including FPR2 [61]. Restoring FPR2 to normal levels and maintaining normal glucose levels shows promise for regulating glucose in diabetes mellitus by modulating neutrophils through RvD1 [4]. Impaired signaling through FPR, resulting from exposure to high glucose levels, leads to paradoxical and detrimental reductions in essential neutrophil trafficking, making diabetic wounds more susceptible to infection [62].

In addition, the pro-inflammatory effects of mitochondrial DAMPs, such as N-formyl peptides, on diabetes mellitus inhibit the citric acid cycle, leading to increased fat oxidation and the release of substantial amounts of ROS [37]. ROS can initiate the NF-κB inflammatory pathway and cause secondary damage to mitochondria [63], as well as high levels of intracellular free fatty acids, which can induce endoplasmic reticulum (ER) stress, disrupt mitochondrial–ER calcium flow, and accelerate cellular necrosis. These DAMPs trigger inflammatory responses and contribute to disease progression [37].

In a comparative analysis of the functional activity of neutrophils in patients with T2D associated with coronary disease, fMLPs induced the release of myeloperoxidase, which increased their peroxidase activity. This was accompanied by a reduction in plasma catalase activity and an increase in lipid peroxidation levels [64].

Finally, the pathophysiological process of T2D may also be regulated by FPR2, which is upregulated in hyperglycemia [58,65]. In this context, FPR inhibitors such as Boc-Phe-Leu-Phe-Leu-Phe (Boc-FLFLF) and Ac-L-Arg-Aib-L-Arg-L-Cα(Me)Phe-NH2 tetrapeptide (UPARANT) seem to be attractive for preventing complications in patients with T2D [66].

### 3.3. Kidney Diseases

Regarding the actions of N-formyl peptides and their receptors in kidney diseases, FPR1 mediates intracellular calcium transport in neutrophils and renal podocytes. It increases the expression of proteins from the MAPK pathway (p-p38, p-ERK, and c-Jun N-terminal kinase (p-JNK), generating glomerular injury and podocyte cell apoptosis [38]. Furthermore, intestinal dysbiosis caused by kidney failure may lead to increased production of N-formyl peptides, which are associated with inflammation and act as agonists of FPR1 in renal tubular epithelial cells [67].

A study showed that patients with kidney disease exhibited increased phenotypic expression of CD11b, CD11c, and CD18 compared to healthy controls in fMLP-induced polymorphonuclear leukocytes, suggesting that the N-formyl peptide has a pathophysiological role in inflammation in CKD [68]. Similarly, another study showed the same pattern in patients undergoing hemodialysis, with fMLP inducing the generation of ROS in polymorphonuclear leukocytes and greater expression of the metalloproteinase receptors CD10 and CD13 compared to controls [69]. On the other hand, AnxA1 has demonstrated protective effects on renal fibrosis in CKD. Renal fibroblasts lacking AnxA1 exhibited elevated alpha-smooth muscle actin (α-SMA) levels and increased collagen 1A1 mRNA expression. However, this increase was mitigated by overexpressing AnxA1 and activating its FPR2 receptor in the cells [70].

In acute kidney injury (AKI), macrophages have a crucial pro-inflammatory role. FPR2 positively regulates this response by increasing the expression of TNF-α, IL-6, and IL-1β in the kidney [71].

### 3.4. Obesity

Adipose tissue can produce N-formyl peptides in response to inflammatory stimuli, worsening chronic low-grade inflammation, insulin resistance, and adipocyte dysfunction. This contributes to obesity-related complications, such as T2D and cardiovascular diseases [49,72,73,74].

In mice with obesity induced by a high-fat diet (HFD), an increase in fMLP levels, particularly within the gastrointestinal tract, coincided with upregulated FPR1 expression, notably in the ileum and colon. Deleting FPR1 in HFD-fed mice reduced fMLP levels in both the blood and intestine, likely due to alterations in the gut microbiota composition that were exacerbated by FPR1 depletion [49].

Obesity-resistant mice (ORM) exhibited lower kinetic parameters of fMLP-induced ROS generation, regardless of inflammation, than control groups. ORM also exhibited increased spontaneous ROS production and impaired Fpr1 and Fpr2 signaling via MAPK pathways, including p38, ERK1/2, and JNK. An HFD altered the roles of FPRs and suppressed MAPK signaling in NADPH oxidase regulation in ORM, potentially affecting their immune function [74].

Higher levels of FPR2 ligands were found in obese adipose tissue compared to lean tissue. Upregulated FPR2 expression was observed in the white adipose tissue of mice with HFD-induced obesity and db/db mice. The systemic depletion of FPR2 in HFD-fed mice reduced body weight, decreased the fat mass percentage, increased the lean mass percentage, and decreased the adipocyte size in white adipose tissue. These effects were mediated by enhanced energy expenditure, possibly through heightened thermogenesis in skeletal muscle. Additionally, FPR2 deficiency improved insulin sensitivity and alleviated the dysregulation of lipid and glucose levels. FPR2-null mice with HFD-induced obesity also exhibited reduced tissue and systemic inflammation by inhibiting macrophage infiltration and macrophage M1 polarization. The authors also noted that FPR2-expressing myeloid cells exacerbated HFD-induced obesity, metabolic disturbances, and inflammation [72].

In another study involving FPR2-null mice, researchers noted a significant increase in age-related weight gain, with a 92% rise in fat mass compared to age-matched WT mice at seven months. Indirect calorimetry suggested that obesity in these mice might be linked to increased food intake, indicating a potential connection to hyperphagia. The absence of the FPR2 receptor resulted in spontaneous obesity, a reduced lifespan, worsened leukocyte dysfunction, and significant interorgan non-resolving inflammation [4].

In obese individuals, neutrophils exhibit increased basal superoxide production, likely influenced by elevated triglyceride levels in the bloodstream. Upon stimulation with fMLP, neutrophils exhibit heightened superoxide production, indicating a primed state in obesity. Additionally, these individuals show higher chemotactic activity, suggesting a potential role in obesity-related disease development. Interestingly, despite the health risks, the ability of neutrophils to combat infections appears unaffected in individuals with a normal weight compared to those with a higher body mass index [75].

### 3.5. Cancer

It has been observed that the expression of FPRs, which are present in the inflammatory context, is associated with tumor progression in several types of cancer [4]. Indeed, FPR1 is highly expressed in glioblastoma cells, and its activation by agonists released from necrotic tumor cells promotes the nuclear translocation of hypoxia-inducible factor 1 alpha (HIF-1α), which increases the protein expression of vascular endothelial growth factor (VEGF), thereby facilitating angiogenesis and subsequent tumor dissemination [76]. Subsequently, researchers found that FPR expressed in glioblastoma cell lines promotes the production of VEGF by tumor cells and transduces signals that selectively induce the phosphorylation of the epidermal growth factor receptor (EGFR). The two receptors interact, intensifying the invasive behavior of the tumor [77].

High expression of FPR1, associated with an aggressive tumor phenotype and poor patient survival, has been observed in gastric cancer [78], neuroblastoma [79], melanoma [80], and astrocytoma [81]. Furthermore, the knockout of the FPR1 gene in mice increased the survival rate in a model of colorectal cancer, and it was also found that the receptor was expressed at significantly higher levels in tumor tissues than in normal tissues [82]. FPR1 also promotes the growth of malignant human hepatoma through the induction of IL-8, which is involved in the angiogenic process [83]. However, a study by Prevete et al. indicates that FPR1 functions as an antitumor agent by inhibiting angiogenesis in gastric cancer, as silencing it promotes tumor proliferation and enhances the response that favors angiogenesis [32].

High FPR3 and FPR2 expression have also been linked to cancer progression. Research by de Paulis et al. indicates that the activation of FPR2 by a peptide derived from *Helicobacter pylori* prompts the migration and proliferation of gastric epithelial cells. Additionally, it has been reported that gastric cancer tissues exhibit high expression of FPR2, activating the MAPK/ERK pathway, which promotes tumor progression and correlates with poor patient survival [84]. Furthermore, it has been suggested that FPR2 could serve as a potential prognostic biomarker and therapeutic target for patients [78].

FPR2 is also highly expressed in colorectal cancer, promoting the prevention of tumor apoptosis and the progression of the invasive process. Silencing with a short hairpin RNA (shRNA) in xenografted mice reduced the expression of the N-cadherin and vimentin proteins [85]. Furthermore, increased FPR2 expression is associated with rapid tumor progression and a worse prognosis for patients with colon and epithelial ovarian cancer [86].

Recent research suggests that the activation of the FPR2 receptor can directly influence glucose metabolism, leading to increased cellular uptake mediated by insulin-sensitive GLUT4 in epithelial carcinoma cells. This process occurs through the indirect activation of cell growth receptors, such as those that respond to insulin-like growth factor, along with the involvement of intracellular pathways associated with cell proliferation and survival [41]. Additionally, signaling triggered by FPR2 inhibits the enzyme that converts pyruvate to acetyl-CoA, preventing it from entering the citric acid cycle and promoting its conversion to lactate, a typical pattern of altered metabolism known as the Warburg effect. This process is linked to the activation of enzymes that control glycolytic metabolism and the stabilization of hypoxia-related factors, contributing to the metabolic reprogramming of epithelial cells [41,42].

It has been suggested that inhibiting FPR3 expression may be a beneficial intervention strategy [40]. Recently, Kim et al. observed that several human tumor cell lines expressed fMet proteins in the mitochondrial and cytosolic fractions of colorectal cancer cells (SW480) [87]. Le Naour et al. observed that dendritic cells from Fpr1-/- mice showed impaired migration in response to chemotherapy-treated colorectal cancer cells. Additionally, these Fpr1-/- mice were highly prone to developing chronic ulcerative colitis and colorectal cancer when exposed to the mutagen azoxymethane followed by dextran sodium sulfate, which induces colitis [88].

### 3.6. Cardiovascular Diseases

Recent studies have highlighted the critical roles of FPRs in the pathogenesis of cardiovascular diseases. The expression of FPRs has been identified in endothelial cells and cardiac macrophages, emphasizing their relevance in cardiac physiology and pathology [32]. Elevated levels of N-formyl peptides in the circulation have been shown to promote endothelial dysfunction and oxidative stress, creating a pro-inflammatory environment [89]. This occurs because FPR1, which is expressed in vascular endothelial and smooth muscle cells, plays a pivotal role in mediating inflammatory responses. It facilitates the recruitment and migration of neutrophils and monocytes to sites of vascular inflammation, a hallmark of atherosclerosis involving endothelial cells. Furthermore, FPR1 has emerged as a potential biomarker for acute myocardial infarction, given its association with inflammatory processes and vascular injury [90]. These findings underscore the therapeutic and diagnostic potential of targeting FPR1 in cardiovascular diseases.

The activation of FPR1 by ligands such as mitochondrial N-formylated peptides promotes pro-inflammatory signaling through the activation of the MAPK cascade (ERK1/2 and p38 MAPK) and subsequent activation of the transcription factor NF-κB, leading to the release of cytokines, including IL-1β, IL-6, and TNF-α [91]. This process is intensified by the production of ROS, mainly via NADPH oxidase, exacerbating endothelial damage and favoring leukocyte recruitment, foam cell formation, and atherosclerotic plaque instability [91].

N-Formyl-methionyl-leucyl-phenylalanine promotes platelet activation by increasing thrombus formation in isolated human platelets under arterial flow conditions. FPR1 deficiency in mice or inhibition in human platelets, achieved through a pharmacological inhibitor, mitigates agonist-induced platelet activation, demonstrating that this receptor plays a role in normal platelet function [92].

In contrast, the activation of FPR2 by pro-resolving ligands such as ANXA1 and lipoxins activates pathways such as p38MAPK, PKC, and Akt, which reduce the expression of pro-inflammatory mediators, increase the release of anti-inflammatory cytokines such as IL-10, promote endothelial integrity, and protect against ischemic injury [91]. FPR2 induces a more stable plaque phenotype due to its effects on smooth muscle cells, thus slowing the progression of atherosclerosis and increasing plaque stability [93].

The binding of the anti-inflammatory ligand AnxA1 to FPR2 promotes the resolution of inflammation by reducing leukocyte recruitment and oxidative stress through the inhibition of NADPH oxidase-mediated ROS production. These mechanisms support the atheroprotective potential of FPR2 activation in vascular inflammation and lesion progression [91,93]. Increasing evidence suggests that AnxA1 may be crucial in alleviating complications associated with ischemia and reperfusion injury (I/RI) [94].

Additionally, in a murine model of inflammatory arthritis, treatment with recombinant human Annexin A1 (hrAnxA1) halted the progression to cardiac diastolic dysfunction, reducing inflammation and myocardial fibrosis, and modulating inflammatory cell infiltration into the heart, suggesting the therapeutic potential of hrAnxA1 in preventing cardiac complications associated with chronic inflammation [95]. However, the roles of FPR2 and its pro-resolving ligand, AnxA1, in cardiovascular disease remains unclear [32].

### 3.7. Neurodegenerative Diseases

The activation of FPR1 and FPR2 by fMLP enhanced the migration of murine NSCs through F-actin polymerization and stimulated their differentiation into neurons [96]. This differentiation was mediated by the phosphatidylinositol 3-kinase (PI3K)-AKT pathway, resulting in ROS generation both in vitro and in vivo [97]. Complementing these findings, the activation of FPR1 has also been shown to promote regenerative processes in neuronal cells. Studies using PC12 cell lines demonstrated that the stimulation of FPR1 with fMLF significantly enhanced cell proliferation, migration, and neurite outgrowth, primarily through ERK1/2 and p38 MAPK pathways, along with the involvement of intracellular calcium mobilization and ROS production [39,98].

Additionally, mitochondrial DAMPs released from disintegrated axonal mitochondria may stimulate murine Schwann cells to extend their cytoplasmic processes, suggesting a mechanism for fMLF-mediated FPR2 activation in the modulation of axonal growth [99]. fMLF-induced chemotactic responses and calcium mobilization in human astrocytoma cell lines demonstrate its ability to activate astrocytes. The secretion of IL-6 in response to fMLF stimulation further underscores this activation, highlighting the functional importance of FPR1 in astrocyte-mediated host defense within the brain [81].

Dysregulated neural stem cell (NSC) activation, driven by abnormal N-formyl peptide signaling, has been linked to the progression of neurodegenerative diseases by fostering neuroinflammation and neuronal dysfunction [39,100].

AD is a complex disorder of the central nervous system (CNS) characterized by the pathological formation of extracellular senile plaques in cerebral tissues, which contain aggregates of the amyloid-β peptide (a cleavage product of the amyloid precursor protein), as well as intracellular neurofibrillary tangles derived from the hyperphosphorylated tau protein in affected neurons [98,101]. Astrocytes and microglial cells in the cortex and hippocampus of mouse models with induced AD exhibited increased expression of FPR1. Furthermore, FPR1 is highly expressed in microglia that infiltrate senile plaques within the brain tissues of AD patients, suggesting that inhibiting FPR activity leads to protective effects on AD [98].

In contrast, FPR2 has been implicated in the pathophysiology of AD through its ability to mediate both the cytotoxic effects of the amyloid-β peptide and the protective effects of the humanin peptide [102]. In an AD mouse model, FPR2 deficiency enhanced cognitive function, reduced tau hyperphosphorylation, and decreased astrocyte activation, suggesting a harmful role of FPR2 in AD pathology [103]. In a study involving an AD mouse model, FPR activation by fMLP impaired spatial memory performance and induced microglia reactivity near plaques, potentially exacerbating neuroinflammation [104]. Mitochondrial-derived DAMP molecules can directly contribute to neuroinflammation in AD. Treatment of human cultured neuronal and microglial cells with mitochondrial lysates resulted in increased levels of TNF-α and IL-8, accompanied by enhanced p38 MAPK phosphorylation and activation of NF-κB, along with reduced levels of its inhibitor, IκBα. This demonstrates that mitochondrial DAMPs, including FPR ligands, can directly induce AD-like neuroinflammatory activity [104].

Parkinson’s disease (PD) is the most common neurodegenerative disorder linked to aging. It is characterized by the degeneration of dopaminergic neurons in the substantia nigra and the formation of Lewy bodies, which are protein aggregates composed of α-synuclein, Parkin, Ubiquitin, and neurofilament [100,101]. The FPR1 agonist fMLF activates microglial cells in human microglia, leading to NOD-like receptor family, pyrin domain-containing 3 (NLRP3) inflammasome activation and the upregulation of the pro-inflammatory cytokines IL-1β, IL-6, and TNF-α. Consequently, this activation triggers the apoptotic death of dopaminergic neurons through the induction of mitochondrial oxidative stress and the activation of pro-apoptotic signaling [39]. Furthermore, it was proposed that gut dysbiosis, reported in early-stage PD, may induce the release of mitochondria-damaging toxins, which subsequently increases DAMP release and initiates neuroinflammation, thereby contributing to PD progression [105].

Myelin abnormalities, oligodendrocyte pathology, and concomitant glial activation characterize multiple sclerosis (MS). In the MS-induced mouse model, the murine cortex showed increased FPR1 expression after treatment. Conversely, depleting FPR1 resulted in a slight yet significant decrease in corpus callosum demyelination, accompanied by reduced astrocyte activation in the cortex and diminished microglia activation in both the corpus callosum and cortex [106]. In the same model, fMLP, known for its potent ability to mobilize neutrophils and stimulate innate immune cells, exacerbated immune reactions in experimental autoimmune encephalomyelitis [107].

## 4. Potential Adjuvant Effects of Bioactive Food Compounds on N-Formyl Peptide Signaling Pathways

Bioactive compounds are naturally present in foods. They contribute to various protective actions and provide health benefits, primarily due to their antioxidant and anti-inflammatory properties [6]. Schepetkin et al. (2016) highlight several natural products, including those derived from medicinal plants, that exhibit inhibitory effects on the signaling pathways mediated by FPR1 and FPR2, suggesting potential antagonistic roles [108]. These compounds may interfere with ligand binding or downstream signaling events, thus attenuating neutrophil chemotaxis, oxidative burst, and the release of inflammatory mediators. This dual capacity of bioactive compounds, acting as agonists or antagonists, depending on the context and target receptor subtype, expands their potential therapeutic applications in modulating excessive inflammation in chronic diseases [108].

In vitro studies have demonstrated that bioactive compounds act to modulate the signaling pathways of N-formyl peptides and their receptors, thereby stimulating neutrophils through FMLP [7,109,110,111]. Bioactive compounds, such as those found in propolis and black poplar (*Populus balsamifera* L.), reduce immune cell activation by controlling calcium mobilization in neutrophils and downregulating FPR1 expression [7]. Furthermore, bioactive compounds in green tea can also inhibit fMLF-stimulated calcium flux and ERK1/2 phosphorylation, leading to a subsequent inhibition of leukocyte migration [112]. For instance, caffeic acid phenethyl ester, a component found in propolis, coffee, and some fruits, decreases neutrophil activation triggered by FMLP, underscoring its anti-inflammatory effects on conditions such as *Helicobacter pylori* infection and other inflammatory diseases [113]. Furthermore, bioactive compounds found in *E. rutaecarpa* (a medicinal plant) regulate FPR expression via the p38 MAPK and JNK pathways. They reduce ROS production and NADPH oxidase activity, highlighting their potential to mitigate oxidative stress and inflammation [110]. Unexpectedly, an in vivo study involving LPS-induced neuroinflammation found that resveratrol activates FPR1 in the striatum and hippocampus, which was associated with reduced neuroinflammation and neuroprotection through the stimulation of IL-10 secretion and the negative regulation of the pro-inflammatory mediators TNF-α and IL-1β [114].

**Figure 5:**
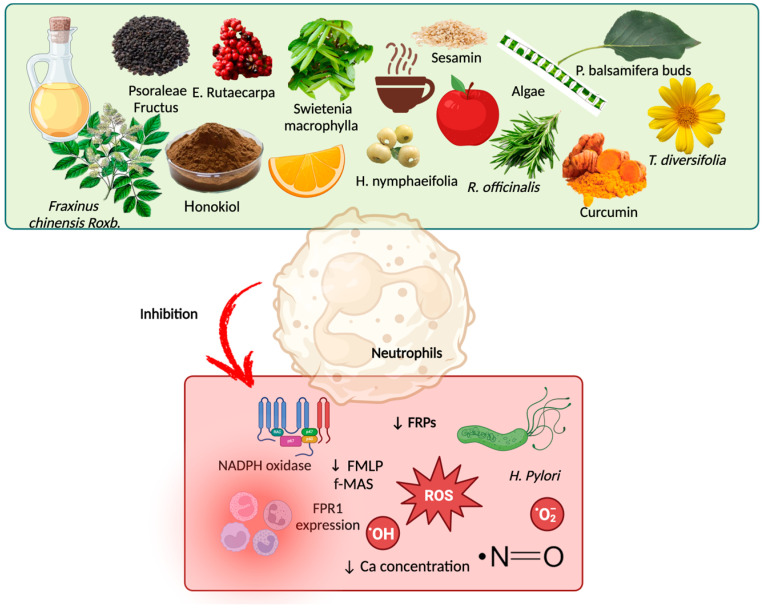
Bioactive compounds as adjuvant treatment for modulation of N-formyl peptide signaling pathways and their receptors. Some bioactive compounds in food may inhibit N-formyl peptides and their receptors, thereby reducing the proliferation of *H. pylori* and the production of reactive oxygen species (ROS) and anion superoxide, as well as mitigating inflammation and reducing the intracellular calcium concentration. Created by BioRender.com.

A few clinical trials have shown the role of bioactive compounds in modulating signaling pathways of bioactive compounds in the regulation of N-formyl peptides [115,116,117]. However, further studies are needed to confirm these effects and their broader implications. Bioactive compounds can regulate N-formyl peptides by inhibiting FPR1 and FPR2, exhibiting competitive blocking or even harmful modulation (Table 1). Figure 5 depicts the mechanisms by which bioactive compounds modulate the signaling pathways of N-formyl peptides and their receptors.

## 5. Final Remarks

N-Formyl peptides activate FPRs, initiating inflammatory pathways governed by the transcription factor NF-κB and the NLRP3 inflammasome. This initiates an inflammatory cytokine cascade that leads to persistent chronic low-grade inflammation, often observed in non-communicable diseases such as kidney disease, obesity, cancer, cardiovascular disease, and neurodegenerative disorders. Furthermore, gut dysbiosis can influence the availability and signaling of N-formyl peptides, forming a triad of dysbiosis, inflammation, and oxidative stress. Consequently, in chronic non-communicable diseases where these three factors intersect, there appears to be a dysregulation in the production of mitochondria- and microbiota-derived N-formyl peptides.

In this context, evaluating biomarkers associated with inflammation, oxidative stress, and the gut microbiota may provide secondary benefits for patients, as monitoring these conditions before disease development could help avoid the need for earlier intervention. In the future, it is anticipated that the assessment of N-formyl peptides and their receptors will serve as a predictive and personalized tool to enhance the medical approach to chronic diseases. It also offers insights into potential treatments that regulate these molecules and their receptors, with a primary focus on their antagonists.

Additionally, nutritional strategies are of paramount importance in the prevention and treatment of chronic non-communicable diseases. Bioactive compounds in foods modulate the behavior of N-formyl peptides, interacting with receptors in a competitive or inhibitory manner, which in turn manage the inflammatory response and oxidative reactions in cells. Therefore, diet and lifestyle become essential allies in disease prevention through modulation of N-formyl peptide signaling pathways and their receptors in a personalized manner.

## Figures and Tables

**Table 1:** Studies investigating the action of bioactive compounds on N-formyl peptides and their receptors.

References	Sample/Study	Intervention	Results
**In Vitro Studies**			
**Wanten et al.** [109]	FMLP-activated neutrophils	Lipid emulsions (fish oil, olive oil, and soya oil)	↓ Cytosolic Ca concentration ↓ Stimulatory effect of FMLP ↓ FMLP-induced cytosolic Ca
**Ko et al.** [110]	FMLP-induced ROS production in neutrophils	Ethanol extract of *E. rutaecarpa* (IC50 μg/mL)	↓ FMLP-induced ROS production ↓ NADPH oxidase activity ↓ LPS-induced NO generation
**Pastene et al.** [118]	Human neutrophils	Apple peel extract (IC50 μg/mL)	↓ Multiplication of two *H. pylori* strains ↓ respiratory burst of neutrophils induced by *H. pylori*, PMA, and FMLP
**Hapner et al.** [119]	Human neutrophils	Lipid-soluble polyphenols (muscadine, curcumin, quercetin, α-tocopherol, and α-tocotrienol)	↓ O_2_^•−^, H_2_O_2_ ↓ FMLP-induced neutrophil oxidative bursts
**Cui et al.** [113]	*E. coli* BL21(DE3)	4.02 µM caffeic acid phenethyl ester	↑ Inhibition of HpPDF ↑ Inhibition of f-MAS
**Chen et al.** [120]	FMLP-activated neutrophils	*Swietenia macrophylla* extract (IC50 μM)	↓ O_2_^•−^ ↓ LPS-induced NO generation
**Schwager et al.** [121]	Granulocyte/macrophage colony-stimulating factor, IL-8, and FMLP	50–1000 mol/L L-ascorbic acid for 4–6 days.	↔ CXCR2 and FMLPR expression ↓ Less cells migrate toward IL-8 and FMLP
**Chen et al.** [122]	FMLP/CB-activated neutrophils	Isoflavone derivatives from *Psoraleae fructus* (IC50 μM)	↓ FMLP/CB-induced elastase activity ↓ O_2_^•−^ ↓ LPS-induced NO generation
**Liu et al.** [123]	Human neutrophils activated by a bacterial FPR1 activator, FMLP	1, 3 and 10 μM honokiol	↓ O_2_^•−^ and elastase levels ↓ FPR1 expression ↔ FPR2 expression ↓ FPR1 agonist-induced Ca mobilization ↓ Phosphorylation of p38 MAPK, ERK, JNK ↓ Mitochondrial N-formyl peptide, fMMYALF, levels
**Tseng et al.** [124]	FMLP/CB-activated neutrophils	Ethyl acetate extract of *K. flaccidum* (IC50 μM)	↓ FMLP/CB-induced elastase activity ↓ O_2_^•−^ ↓ LPS-induced NO generation
**Chang et al.** [125]	FMLP/CB-activated neutrophils	*Fraxinus chinensis* Roxb. (Oleaceae) extract (IC50 μM)	↓ O_2_^•−^ ↓ FMLP/CB-induced elastase activity ↓ LPS-induced NO generation ↑ MAPKs and IκBα expression
**Lai et al.** [126]	FMLP/CB-activated neutrophils	Compounds isolated from the root bark of *H. nymphaeifolia* (IC50 μM)	↓ O_2_^•−^ ↓ FMLP-induced elastase release ↓ LPS-induced NO generation
**Schepetkin et al.** [7]	Human neutrophils preincubated for 10 min with 5 nM FMLP or WKYMVM	3.1, 6.33, 12.5, or 25 µL/dL essential oils extracted from P. balsamifera buds, propolis, and pure nerolidol	↓ FMLP-induced neutrophil chemotaxis ↓ Activation of FPR1 and FPR2
**In Vivo Studies**			
**Calvello et al.** [114]	129SV male mice (LPS-induced neuroinflammation)	50 mg/kg resveratrol for 10 days	↓ IL-1β and TNF-α gene and protein expression in the striatum and hippocampus ↑ FPR1 and SIRT1 expression and IL-10 levels
**In Vitro and In Vivo Studies**			
**Zhu et al.** [3]	In vitro: human monocyte cell line THP-1 In vivo: KM mice (murine air-pouch model)	In vitro: from 5 to 500 μM green tea polyphenol-epigallocatechin-3-gallate for 24 h In vivo: 20 mg/kg/day of green tea polyphenol-epigallocatechin-3-gallate for 2 days	In vitro: ↓ FMLP-induced leukocyte cell migration, ↓ FMLP stimulation of Ca flux ↓ Phosphorylation of ERK1/2 ↓ FPR-mediated leukocyte migration In vivo: ↓ fMLP-induced leukocyte migration
**Cui et al.** [127]	In vitro: human monocyte cell line THP-1 In vivo: C57BL/6 mice (murine air-pouch model)	In vitro: sesamin (12.5 and 50 μmol/L) In vivo: intraperitoneal administration of sesamin (12 mg·kg^−1^·d^−1^) for 2 days	In vitro: ↓ FMLP-induced chemotaxis ↓ FMLP-induced nuclear factor-κB activation ↓ FMLP-induced ERK1/2 phosphorylation ↔ FMLP-induced calcium flux In vivo: ↓ leukocyte infiltration into the air pouch induced by FMLP
**Silva et al.** [117]	In vitro: peritoneal neutrophils In vivo: male Wistar rats	In vitro: 1, 10, or 100 μg/mL *R. officinalis* extract In vivo: 100, 200, or 400 mg/kg *R. officinalis* extract orally for four hours	In vitro: ↓ neutrophil chemotaxis, NO production ↓ shedding of L-selectin and β2 integrin expression ↓ FMLP-induced migration In vivo: ↓ SOD, TBARS, LTB4, PGE2, IL-6, and TNF-α levels
**Broering et al.** [128]	In vitro: LPS-stimulated neutrophils In vivo: male Swiss mice (carrageenan-induced inflammation)	In vitro: *T. diversifolia* extract (1, 10, or 100 μg/mL) In vivo: pretreatment one hour before with *T. diversifolia* extract (0.1, 1, or 3 mg/kg) orally	In vitro: ↓ TNF, IL-1β and IL-6 levels, neutrophil chemotaxis, and NO production ↔ levels of CXCL-1 ↓ FMLP-induced migration In vivo: ↓ leukocyte migration, ↓ connective tissue edema ↓ TNF, IL-1β and CXCL-1 levels ↔ IL-6 levels
**Human Studies**			
**Charman et al.** [115]	RCT: 22 subjects before cardiac surgery Ex vivo: neutrophil activation by superoxide anion generation	RCT: 8 g/day fish oil (omega 3) for 6 weeks	RCT: ↓ plasma VLDL and TG levels ↑ HDL cholesterol levels, ↓ troponin levels ↔ generation or myeloperoxidase release Ex vivo: ↔ FMLP-stimulated neutrophil O_2_^•−^ levels
**Imhof et al.** [116]	42 healthy subjects/RCT	Ethanol (concentration 12.5%), beer (5.6%), red wine (12.5%), or dealcoholized beverages for 3 weeks	Ethanol: ↓ MCP-1-stimulated migration and FMLP levels, ↓ ICAM-1 levels, ↓ TNF-α levels Dealcoholized red wine: ↓ MCP-1-stimulated migration and FMLP levels, ↓ ICAM-1 levels, Red wine: ↑ HDL-C levels, ↑ selectin levels, ↓ ICAM-1 levels Dealcoholized beer: ↑ TNF-α levels

Abbreviations: Ca, calcium; CB, cytochalasin; CXCR2, C-X-C motif chemokine receptor 2; CXCL, chemokine (C-X-C motif) ligand; ERK, extracellular signal-regulated kinase; fMLP/FMLP, N-formylmethionyl-leucyl-phenylalanine; fMMYALF, N-formylmethionyl-leucyl-alanine-leucine-phenylalanine; FPR1, formyl peptide receptor 1; FPR2, formyl peptide receptor 2; HDL-C, high-density lipoprotein cholesterol; ICAM-1, intercellular adhesion molecule 1; IκBα, inhibitor of kappa B alpha; IL, interleukin; JNK, c-Jun N-terminal kinase; LTB4, leukotriene B4; LPS, lipopolysaccharide; MAPK, mitogen-activated protein kinase; MCP-1, monocyte chemoattractant protein-1; PGE2, prostaglandin E2; RCT, randomized controlled trial; ROS, reactive oxygen species; SOD, superoxide dismutase; TBARS, thiobarbituric acid-reactive substances; TNF-α, tumor necrosis factor alpha; VLDL, very-low-density lipoprotein; TG, triglyceride; O2^•−^, superoxide anion; NO, nitric oxide; H_2_O_2_, hydrogen peroxide; ↓ decrease; ↑ increase; ↔ no changes

## Data Availability

No new data were created or analyzed in this study. Data sharing is not applicable to this article.

## Abbreviations

10-fTHF	10-Formyltetrahydrofolate
AD	Alzheimer’s disease
AKI	Acute kidney injury
ANXA1	Annexin A1
Ca	Calcium
CB	Cytochalasin
CKD	Chronic kidney disease
CNS	Central nervous system
CVD	Cardiovascular disease
CXCL	Chemokine (C-X-C motif) ligand
CXCR2	C-X-C motif chemokine receptor 2
DAMPs	Damage-associated molecular patterns
EGFR	Epidermal growth factor receptor
ERK	Extracellular signal-regulated kinase
ERK1/2	Extracellular signal-regulated kinase 1/2
F-ACTIN	Filamentous actin
FMIT	Mitochondrial N-formyl peptides
FMT	Formyltransferase
FPRs	Formyl peptide receptors
fMet	N-Formylmethionine
fMIFL	N-Formyl-methionine-isoleucine-phenylalanine-leucine
fMIVIL	N-Formyl-methionine-isoleucine-valine-isoleucine-leucine
fMLFK	N-Formyl-Met-Leu-Phe-Lys
fMLP	N-Formyl-methionyl-leucyl-phenylalanine
fMMYALF	N-Formyl-methionine-methionine-tyrosine-alanine-leucine-phenylalanine
fMLKLIV	N-Formyl-methionine-leucine-lysine-leucine-isoleucine-valine
fMYFINILTL	N-Formyl-methionine-tyrosine-phenylalanine-isoleucine-asparagine-isoleucine-leucine-threonine-leucine
FMLP/CB	Formyl-L-methionyl-L-leucyl-L-phenyl-alanine/cytochalasin B
fMLP/FMLP	N-Formyl-methionyl-leucyl-phenylalanine
FPR1	Formyl peptide receptor 1
FPR2	Formyl peptide receptor 2
GPCRs	G protein-coupled receptors
GLUT	Glucose transporter
HO1	Heme oxygenase-1
H_2_O_2_	Hydrogen peroxide
HDL-C	High-density lipoprotein cholesterol
HFD	High-fat diet
HIF-1α	Hypoxia-inducible factor 1 alpha
IL	Interleukin
IL-6	Interleukin-6
I/RI	Ischemia–reperfusion injury
ICAM-1	Intercellular adhesion molecule 1
IECs	Intestinal epithelial cells
IκBα	Inhibitor of kappa B alpha
JNK	c-Jun N-terminal kinase
LPS	Lipopolysaccharide
LXA4	Lipoxin A4
LL-37	Leucine-leucine-37
LTB4	Leukotriene B4
MAPK	Mitogen-activated protein kinase
MCT-2	Mitocryptide-2
MDA	Malondialdehyde
MetAP	Methionine aminopeptidase
MAPKs	Mitogen-activated protein kinases
MCP-1	Monocyte chemoattractant protein-1
mPTP	Mitochondrial permeability transition pore
MS	Multiple sclerosis
MUC	Mucin
NADPH	Nicotinamide adenine dinucleotide phosphate reduced
NF-κB	Factor nuclear kappa B
NQO1	NAD(P)H quinone dehydrogenase 1
Nrf2	Nuclear factor erythroid 2-related factor 2
NLRP3	Pyrin domain-containing 3
NLRs	NOD-like receptors
NOX	Nitrogen oxide
NSCs	Neural stem cells
O_2_^•−^	Superoxide anion
ORM	Obesity-resistant mice
PAMPs	Pathogen-associated molecular patterns
PDF	Peptide deformylase
PSM	Phenol-soluble modulins
PSMα	Modulins of the alpha type
MAPK	p38 mitogen-activated protein kinase
PD	Parkinson’s disease
PGE2	Prostaglandin E2
PI3K-AKT	Phosphoinositide 3-kinase/Protein kinase B
PMA	Phorbol myristate acetate
RCT	Randomized controlled trial
ROS	Reactive oxygen species
RvD1	Resolvin D1
shRNA	Short hairpin RNA
SIRT1	Sirtuin 1
SOD	Superoxide dismutase
TNF-α	Tumor necrosis factor alpha
T2D	Type 2 diabetes mellitus
TBARS	Thiobarbituric acid-reactive substances
TGs	Triglycerides
VEGF	Vascular endothelial growth factor
VLDL	Very-low-density lipoprotein
WKYMVM	Trp-Lys-Tyr-Val-Met
WT	Wild-type
α-SMA	alpha-Smooth muscle actin
